# When “Good Evidence” Is Not Enough: A Case of Global Malaria Policy Development

**DOI:** 10.1002/gch2.201700077

**Published:** 2018-03-22

**Authors:** Bianca J. D'Souza, Justin O. Parkhurst

**Affiliations:** ^1^ Department of Global Health and Development London School of Hygiene and Tropical Medicine Keppel Street London WC1E 7HT UK; ^2^ Department of Health Policy London School of Economics and Political Science Houghton Street London WC2A 2AE UK

**Keywords:** evidence, governance, legitimacy, policy process, WHO

## Abstract

This paper presents findings from a case study of two different policy development processes within the WHO's malaria department. By comparing the policy processes for the interventions of intermittent preventive treatment in infants versus children, the findings suggest that “good evidence” from a technical perspective, though important, is not sufficient to ensure universal agreement and uptake of recommendations. An analysis of 29 key informant interviews finds that evidence also needs to be relevant to the policy question being asked, and that expert actors retain a concern over the legitimacy of the process by which technical evidence is brought to bear in the policy development process. Previous findings from the field of sustainable development, that evidence must be credible, salient, and legitimate to be accepted by the public, appears to apply equally within scientific advisory committees. While the WHO has principally focused on technical criteria for evidence inclusion in its policy development processes, this study suggests that the design and functionality of its advisory bodies must also enable transparent, responsive, and accepted processes of evidence review to ensure that these bodies are effective in producing advice that engenders change in policy and practice.

## 1 Introduction

This paper presents findings from a study investigating evidence use in global malaria policy development at the World Health Organization (WHO). Past work looking at decision making at WHO has engaged with topics such as its criteria for guideline development,^1^ or critical reflection on the organization's response to global health crises.^2^ Here, however, the focus is not so much on the outcomes of decisions, but rather on the internal processes involved—observing what is sometimes referred to as the “black box” of how evidence actually informs the policy process,^3^ within the primary global health institution responsible for the production of normative guidance to 193 member states.^4^

The use of evidence has been a long established part of the policy process, and within public health, research evidence is widely considered as the necessary foundation for many health policy decisions.^5^ However, many have argued that the implied linear process between the knowledge produced by researchers and the policies developed by policy makers oversimplifies and does not adequately account for the complexities and political nature of policy making.^6^

There is already a substantial body of work focused on the use of evidence in policy.[[qv: 5c,7]] Many works concerned with “uptake” of research findings have attempted to identify ways to overcome “barriers” and increase “knowledge transfer”.^8^ Yet, numerous scholars have also drawn attention to the shortcoming of these approaches, including how they tend to exclude political considerations from policy decision making.[[qv: 6b,9]] The public health community, it has been argued, has to consider how to move beyond simple notions of barriers and facilitators or a “more is better” approach.[[qv: 6b,9]] Parkhurst,[[qv: 6b]] for instance, argues that a shift is needed to engage with questions of what improved evidence use looks like by asking explicitly normative questions about how we might judge “good evidence” in terms of policy appropriateness, and the “good use of evidence” from a perspective of the decision making process. These considerations can then enable reflections on how to improve “evidence advisory systems” over time, rather than simply focusing on uptake of single pieces of research.[[qv: 6b]]

Scientific advisory committees within technical agencies (such as WHO) could be seen in many ways as archetypal technocratic agencies within such evidence advisory systems—made up of experts that are explicitly tasked with review of scientific information. In the case of the WHO Global Malaria Program (WHO‐GMP), the focus of this study, scientific advisory committee members are tasked with reviewing the evidence and advising WHO in their development of global policy recommendations to control and eliminate malaria.^10^

There is also a growing literature providing insights into the role and function of scientific advisory committees. Many of these are concerned with how to improve their inner workings in one way or another, for example by including patient experience information,^11^ or economic information,^12^ in order to promote the integration of evidence into health policy and practice. Other literature has been concerned with exploring how such bodies deal with constructing or facilitating a process less prone to bias, for example by applying clear, comprehensive, and consistent evidence inclusion criteria.^13^

What many of these studies have in common is their focus on advisory bodies serving national governments. In health care, an exemplar often referenced is the National Institute for Health and Care Excellence (NICE) in England and Wales, which serves a mandated role to develop guidelines and make decisions that can have direct influence over policy and practice for the National Health Service. Yet, few studies examine the processes and perceptions of global health scientific advisory committees, which advise institutions such as the WHO. This may be an important distinction, however, because global health governance systems are decidedly different to national bodies, given the lack of a supreme authority and much more indirect systems of accountability to population groups.

This paper focuses on WHO‐GMP, as an example of an international policy and guidance producer, and presents the findings from a case study of two different policy development processes for malaria control and prevention that took place within the department between 2006 and 2012. Both policies relate to what is known within the global malaria community as “intermittent preventive treatment”, or IPT, which is the delivery of a treatment dose of an antimalarial drug given at prespecified times for the prevention of malaria, regardless of the presence of symptoms or confirmed malaria infection. The two policy development processes that are compared are for the policies for IPT in infants (IPTi) versus in children (IPTc – now known as Seasonal Malaria Chemoprevention or SMC). Although there are commonalities between the two policies, the two policy development processes that led to them resulted in two very different perceptions by stakeholders about the success of those processes. For IPTi,^14^ the process through which evidence was used to inform policy was contentious and considered less than ideal to those who were involved.^15^ In comparison for SMC,^16^ the process was viewed by those involved as a model of efficiency.^17^

By comparing the negative perception of one process in relation to the positive assessment of the other, however, this paper aims to explore some of the key features and influences shaping the use of evidence to inform policy decisions according to key stakeholders who serve within this technical body advising on global health guidelines.

## 2 Data Collection and Analysis

Data for this analysis came from 29 key informants interviewed between October 2014 and October 2015. The interviews were semi‐structured and sampling was purposive to ensure a wide range of perspectives from those involved in the IPTi and/or SMC policy processes. They included: (a) staff from the Bill & Melinda Gates Foundation (BMGF), funders of the IPTi and SMC studies; (b) staff from the research institutions who conducted the IPTi and SMC studies; (c) members of two of WHO‐GMP's scientific advisory committees—the Chemotherapy Technical Expert Group (TEG) and the Malaria Policy Advisory Committee (MPAC)—who advised WHO‐GMP on the IPTi and SMC policies; and (d) staff from WHO‐GMP responsible for issuing the IPTi and SMC policies to relevant member states.

Data also included published and unpublished documentary sources, including official policy documents for IPTi and SMC, scientific advisory committee meeting reports for IPTi and SMC, and internal BMGF and WHO‐GMP documents on IPTi and SMC. Observational notes documented during meetings and conferences between March 2011 and October 2015 were also considered, as supplementary to the interview and document analysis. Data was organized and analyzed with the use of the Nvivo10 software package. Results were analyzed thematically, with no strict boundaries between data collection and analysis, as some themes began to emerge during the course of data collection. The interviews produced multiple narratives, which sometimes complemented or contradicted each other, but collectively provided insights into evidence use and the policy process from the point of view of the participants in it, which was the purpose of this interpretive case study.

The broad starting framework for analysis was derived from a study by Cash and colleagues,^18^ conducted in the field of environmental sustainability. The authors found that the effectiveness of science to inform policy rested on three key factors: credibility, which refers to the scientific adequacy of the evidence; salience, which refers to the relevance of the science to the needs of decision‐makers; and legitimacy, which refers to the perception that the evidence generation and use has been unbiased and fair in its treatment of divergent stakeholder interests. Parkhurst,[[qv: 6b]] similarly draws on this work to discuss the concepts of “good evidence” for policy or the “good use of evidence” within policy processes. “Good evidence” in this work is taken to capture evidence which is both appropriate to specific decisions being made (reflecting salience), but also of high quality according to principles of scientific good practice (often espoused by champions of so‐called evidence based, or evidence informed, policymaking). “The good use of evidence” for policy, however, is presented by Parkhurst as capturing multiple concepts of legitimacy—including input legitimacy (decisions made by authorized representatives of the public); output legitimacy (decisions that achieve their intended goals to serve the public); and throughput legitimacy (decision processes themselves judged legitimate by beneficiaries). These broad concepts related to credibility, salience, and legitimacy, then allowed exploration of data to consider how similarities and differences might be seen between the two policy processes studied—in terms of features of the evidence base, its relevance to needs, and the process by which the evidence was used.

## 3 Findings

### 3.1 A Tale of Two Processes

Malaria is a complex, mosquito‐borne, infectious disease, and a major global public health problem. In 2015 there were over 200 million new cases of malaria and nearly 500 000 deaths.^19^ An estimated 90% of malaria cases and 92% of malaria deaths occur in Africa, the majority among children below five years of age.^19^ This makes this particular age group in this particular geographical location an important target for global health policy makers and funders of public health research and programs who have a vested interest in reducing the global burden of malaria for moral, economic, and global health security reasons.^20^

According to many in the global malaria community, the late 1990s marked a turning point in global interest in malaria.^21^ There was a resurgence of international attention for the disease after what was perceived to be the relative failure of the malaria eradication campaign of the 1960s.[[qv: 21b]] Over the following decades, the malaria agenda went from the grand aspiration of eradication to a period of neglect to what is once again a recovered and enthusiastic vision of “accelerating toward elimination”, which is the goal of WHO's 2016–2030 global strategy for malaria.[[qv: 20,21b]]

The resurgence in attention was accompanied by a huge rise in the funds available for malaria research, control, as well as advocacy. This is reflected in the creation of Multilateral Initiative on Malaria in 1997, the Roll Back Malaria Partnership in 1998, BMGF in 1999, and the Global Fund against HIV/AIDS, Tuberculosis and Malaria in 2001.^22^ The increase in funding, particularly from the BMGF,^23^ provided new opportunities for research for increasing numbers of researchers, and it led to greater discussion among researchers around how few interventions against malaria existed.^24^ At the end of the 1990s there were limited tools for malaria treatment and control, but that would soon change.[[qv: 21b]]

In 2001, the results of a randomized controlled trial (RCT) in Tanzania using Intermittent Preventive Treatment in infants (IPTi) employing the antimalarial drug Sulphadoxine–Pyrimethamine, delivered through the Expanded Program on Immunization, showed that this could be a useful intervention as it reduced clinical malaria episodes in infants by 59%.^25^ This generated much enthusiasm among the core group of scientists involved in the trial, and subsequently in the medical profession,^26^ because the results were considered potentially game‐changing compared to the 35% pooled protective efficacy of malaria prevention interventions in pregnancy, i.e., Intermittent Preventive Treatment in pregnant women (IPTp) and insecticide‐treated mosquito nets (ITNs).^27^ The researchers involved along with researchers from other institutions, and staff at WHO and UNICEF, subsequently formed a cross‐institutional BMGF‐funded global research partnership in 2003—the IPTi Consortium—that declared that they had “developed a research and implementation agenda that will rapidly resolve the outstanding scientific questions about this innovative form of malaria control, and move the intervention into policy and practice” within five years, by the end of 2008.^28^ They also added, somewhat ambitiously, that they had “prepared a strategic plan showing how, by the end of 2005, sufficient information will exist on which to base a policy recommendation.”^28^

As part of the strategic plan and policy goals of the IPTi Consortium, a concurrent Policy Platform was established in WHO‐GMP in 2005 to review the evidence gathered through the Consortium's research groups.^29^ Its role was to prepare evidence as it became available from the IPTi studies for a WHO technical review, so that WHO‐GMP could reach a global recommendation on IPTi. This technical review involved the assessment of evidence by a series of WHO scientific advisory committees—a TEG, a Technical and Research Advisory Committee (TRAC) that reviewed TEG recommendations, and a Strategic and Technical Advisory Group (STAG) that reviewed TRAC recommendations.

For IPTi, the first TEG meeting was held in October 2006 and assessed the results of 11 studies on the efficacy and safety of IPTi in infants and children.^30^ At the time of the 2006 review, three of the trials on efficacy and safety were not published. The recommendation of the 2006 TEG to WHO was for countries to implement IPTi alongside rigorous monitoring, and if as additional data on IPTi emerged, there would be further assessments of the intervention. This TEG recommendation went to the TRAC in December 2006 where it was endorsed. The final level of review, before going to the WHO Director General, was at the STAG due to be held in May 2007. However, WHO cancelled this meeting and decided that a second TEG should be convened. This decision was triggered by newly available results of the outstanding trials released early in 2007, which reported the occurrence of severe adverse reactions that had not been reported in previous trials. In October 2007 a second meeting of the TEG took place, recognizing IPTi was a “promising intervention” but they recanted their previous recommendation and, to be cautious, suggested another review be held in 2008 when new data became available.^31^ The deliberations of the TEG were negatively perceived by some IPTi researchers as unnecessary delays in the evidence advisory process, and led to increasing frustration within the IPTi Consortium.^15^ This led to increasing tensions both amongst the researchers, and with WHO‐GMP and its TEG, over differences in perceptions of time urgency, the meaning of rigorous evidence review, and the role of scientists.^15^ In an attempt to drive what was perceived to be a circular and slow moving process forward, the BMGF decided to commission an independent study from the U.S. Institute of Medicine (IoM) in mid‐2007 to evaluate the IPTi results. This process, however, was viewed by multiple individuals interviewed as being at best irritating and at worse undermining to WHO‐GMP. In July 2008, the IoM review concluded that IPTi was “worthy of further investment” and was potentially “ready to move to a new level,” implying program implementation in countries where IPTi would be effective.^32^ It is difficult to say whether the 2008 IoM conclusion had any bearing on WHO‐GMP (interviewees suggested it did not) but in April 2009, eight years after the first IPTi study was published, a final meeting of the TEG judged the IPTi evidence base to finally be sufficiently acceptable, and endorsed a global policy recommendation on IPTi by WHO to member states.^33^

The political fall‐out from the perceived delays and tensions in the IPTi policy process was among the factors that precipitated WHO‐GMP to review its many existing policy setting mechanisms in what by that point was an increasingly competitive global health policy environment for WHO‐GMP.^34^ Specifically, in 2011, WHO‐GMP embarked on a policy setting strengthening exercise to increase the timeliness, transparency, independence, and relevance of its recommendations to WHO member states in relation to malaria control and elimination.^35^ The result was the scientific advisory committee, MPAC, first convened in 2012, to provide “independent strategic advice and technical input to the WHO for the development of policy recommendations covering all aspects of malaria control and elimination.”^35^

The first body of evidence to come under this new system of MPAC review was for SMC. SMC is defined as the intermittent administration (once a month, up to four months) of full treatment courses of an antimalarial medicine (Amodiaquine + Sulphadoxine–Pyrimethamine) to children under the age of five during the malaria season to prevent malarial illness by maintaining therapeutic antimalarial drug concentrations in the blood throughout the period of greatest malarial risk.^16^

Research on SMC had been going on for several years before the MPAC was formed. As in the case of IPTi, enthusiasm for SMC was based on positive findings from a RCT, but in Senegal instead of Tanzania, also published in the Lancet, but in 2006 instead of 2001, in this case showing an even higher 86% protective efficacy, compared to the 59% protective efficacy of the first IPTi RCT.^25^, ^36^ More notably, however, unlike with the previous case, an official consortium with an overt agenda to achieve policy goals was never formed, and there appeared to be little tension between actors involved in the evidence advisory process. Instead, a series of informal collaborative meetings between SMC researchers and WHO with relevant national policy makers and program managers to identify outstanding priorities for research relevant to a SMC policy decision took place in 2008.^37^ These were followed by several large‐scale evaluation studies in 2009.^38^ Meanwhile, there were periodic informal reviews of the evidence dossier by experts to ensure that the necessary information was being collated for an informed decision by policy makers.^37^ This culminated in a single formal meeting of the TEG to review the evidence for SMC in May 2011, which resulted in a unanimous positive recommendation for the intervention despite the lack of an implementation mechanism.^39^ The recommendation was reviewed by the newly formed MPAC in February 2012, and by March, six years after the first SMC study was published, WHO‐GMP issued the policy recommendation for SMC.^16^

**Figure 1:**
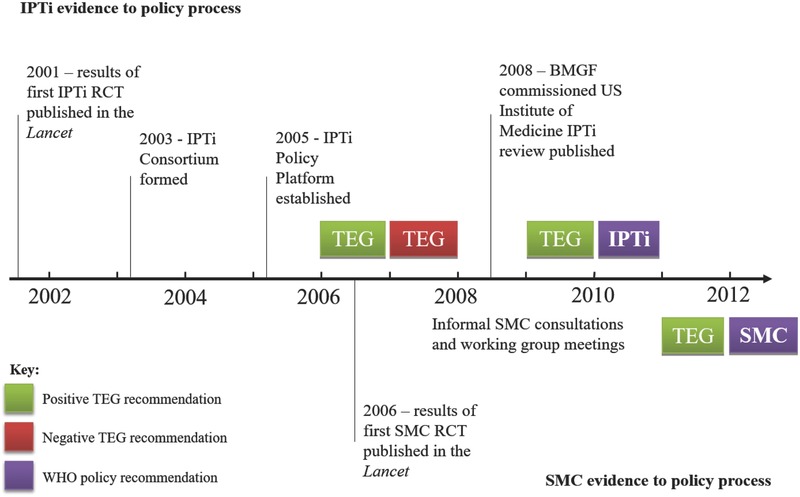
A simplified timeline of the evidence to policy process for two forms of intermittent preventive treatment (IPTi and SMC).

Although the overall timeline between initial results publication to an eventual policy recommendation by WHO‐GMP had some similarities for both IPTi and SMC **(Figure** 1 **)**, as described earlier, many stakeholders viewed the policy development process for SMC as considerably better—a “model” process^17^—to that for IPTi. The reasons for why appear to relate to both features of the evidence itself as well as perceptions of the policy process, explored next.

### 3.2 Strength and Quality of Evidence (Credibility)

Although there were several questions about the efficacy of IPTi (such as the extent to which IPTi merely delayed the onset of malaria and how much that mattered, or the impact of increasing drug resistance to Sulphadoxine–Pyrimethamine in parts of East Africa), the main criticism of several interviewees regarding the nature of the evidence was that the positive results from the first IPTi trial were not reproduced to the same high levels in later trials—the pooled protective efficacy of IPTi was 30%,^14^ compared to the 59% protective efficacy from the first trial,^25^ which is to say that IPTi trials subsequent to the first one showed much lower protective efficacy on average. For some, this raised questions about the benefits of IPTi:

*One of the big issues with IPTi was that the evidence didn't all point in the same direction. So the decisions were, you know, I think it was harder for people to have the level of confidence in them that they might have had with SMC where there's not much evidence going in the other directions. – KI41*

Heterogeneity was not an issue for the SMC set of studies, where the pooled protective efficacy of the intervention was 75%,^40^ compared to the 86% protective efficacy from the first trial,^36^ which is to say that all SMC trial results showed similarity with consistently high protective efficacy.^39^, ^40^

Many interviewees seemed to assume this consistency between SMC trial results reflected strength of the results, which in turn might have helped the evidence base for SMC appear of higher quality compared to IPTi. However, the inconsistency in IPTi trial results is not necessarily a sign of weakness or lower quality, as the difference can be due to features of the study environments. The SMC studies all took place within a narrow geographic band of West Africa with similar and highly seasonal transmission (60% of cases occurring within four months of the year). In contrast, IPTi trials took place all over sub‐Saharan Africa in a variety of transmission and epidemiological settings (which is common for many malaria interventions). Therefore, it would have been expected that any given trial would show higher protective efficacy, and greater consistency, when tested in more narrow trial regions (although the absolute level would depend of course on features of the intervention, including the drugs used).

In addition, the protective efficacy of IPTi is not dissimilar to other preventative malaria interventions widely recommended; for example, the best known preventive intervention against malaria, ITNs, has a protective efficacy of 55% in children.^27^ The complexity of preventing a complicated disease in a wide variety of (and ever‐changing) epidemiological settings is the reason no “magic bullet” exists in malaria control and why high coverage of a mix of interventions that is most suited to local transmission patterns is recommended by WHO.^20^ So when it comes to protective efficacy as a proxy measure of the strength of an evidence base, it could be said that SMC is more of an outlier for preventive malaria interventions, given its consistency but also relatedly, the narrow geographic focus of studies. When thinking about the IPTi case in retrospect, many interviewees conceded this point, but opened up as to other reasons why they found the SMC evidence base to be relatively stronger and more credible.

### 3.3 Policy Relevance (Salience)

The perception of higher “strength” for SMC might have been compounded by the fact that the SMC study sites in the intervention region of Africa were also the proposed implementation sites for the SMC policy, which resulted in an unusual situation for the scientific advisory committees (TEG and MPAC) that systematically reviewed the evidence base on SMC in order to advise WHO‐GMP on a policy recommendation. In many other cases, these bodies need to deliberate about the applicability of study findings from a wide range of settings to the target contexts. Yet with SMC, because the study region was the implementation region, the evidence base reviewed had both high internal and external validity, which as several interviewees pointed out, made making a positive policy recommendation an easy choice and a relatively straightforward process compared to IPTi. Whereas in comparison the TEG for IPTi (MPAC did not exist at the time) had far more nuances to consider in its systematic review of the evidence available at the time.^41^

For example, IPTi was sometimes described as “the wrong drug… at the wrong time,”^42^ even though in reality, the programmatic feasibility (implementation) of IPTi was recognized as being extremely important by the IPTi Consortium.^43^ Unfortunately, this did not appear to be enough. WHO‐GMP and some other interviewees were uncertain as to how IPTi could be implemented and monitored in view of the increasing drug resistance to Sulphadoxine–Pyrimethamine in some parts of Africa and the lack of capacity in some countries, particularly at district level, to monitor levels of drug resistance in order to know where best to target the drug (making the drug essentially ineffective in those areas, hence the view that it might be the “wrong drug”). In addition, the actual relevance of IPTi was also questioned in countries where the coverage of its delivery mechanism, the Expanded Program on Immunization, was low, or where there was highly seasonal malaria transmission (which is to say the delivery of the drug would not in some areas of countries be coinciding with the expected peaks in the number of malaria cases, hence the view that it might be delivered at the “wrong time”), as IPTi would have a very small effect.^44^ Although these issues were not specific to IPTi, WHO guidelines had to take into account local heterogeneity of countries' epidemiological profiles and the need to disaggregate their policy to sub‐national levels. This was less of an issue for the SMC policy consideration, as there was epidemiological homogeneity for the reasons described earlier, and because the policy would only apply to certain parts of certain countries where 60% of cases occurred within four months of the year, the policy in some ways was already disaggregated to sub‐national levels.

SMC, in comparison to IPTi, was also described as having higher “practicability” and “generalizability” beyond just a research setting. This also seemed to contribute to its evidence base's perceived “strength” and salience. As one member of MPAC described:

*I think the evidence base for SMC is pretty strong. I mean there are a number of really quite convincing and sufficiently large studies that show major impact. I mean you're always concerned with, I think, a number of things; one is the size of the studies, the consistency of the results, and the scale of impact, and that's the first step. Obviously you're then concerned about the practicability, because there it's quite possible to have an intervention, which is in a controlled setting, demonstrably effective, but it may simply not be practical. I think SMC has the advantage of firstly, it's got a good evidence base; the studies [have] sufficient numbers, are sufficiently large, and showing really major impact, and certainly some of the studies have been conducted under conditions which would allow you to already extend it to the idea that this could be applied in a [real‐life] setting rather than a small‐scale research study. – KI34*

The reasons for the difference in generalizability are varied, and among the explanations that were offered by interviewees was the difference in age group and banding (infants versus children), and the study location (highly seasonal transmission versus a variety of transmission settings). The SMC studies were focused only in areas of highly seasonal transmission whereas the goal of the IPTi studies was to be generalizable to all of Sub Saharan Africa, which has far more variability in malaria transmission, sometimes within the same country. This, in hindsight, made generalizability difficult due to the variability in results, compared to the relative homogeneity of the SMC study results due to the homogeneous transmission settings.

In short, by conducting the SMC RCTs in the countries where the intervention, if successful, would be eventually rolled out, SMC researchers helped ensure that their portfolio of research answered a wide enough range of useful questions to policy makers that it was considered more relevant compared with IPTi. This is despite SMC having some perceived implementation‐related weaknesses such as no single pre‐existing delivery mechanism. For example, IPTi delivery via the Expanded Program on Immunization was viewed by many as a potential strength, as it meant delivery would be through the existing health system, when most mothers were already visiting health clinics with their infants for their WHO‐recommended vaccination schedule. Some interviewees, however, perceived the lack of a single pre‐existing delivery mechanism as a potential strength for SMC, rather than a critical weakness, as to them it meant that national malaria control programs could have more flexibility and control over how the intervention could best be delivered in their local context.

### 3.4 Legitimacy of the Process

A final theme explored was features related to the perceived legitimacy of the two processes, and how this may help to explain why interviewees saw the SMC process as better than that for IPTi. At the time of the IPTi Consortium, the evidence review process at WHO‐GMP involved the assessment of evidence by a series of scientific advisory committees—the TEG, TRAC, and STAG.^34^ By the time of evidence review for the SMC studies in 2011, however, a restructure intended to make the policy process more “transparent, responsive, and credible,”^35^ meant there were two levels, the TEG and the MPAC, which the TEG reported to. Beyond this, however, two further sub‐themes emerged related to the legitimacy of the processes.

#### 3.4.1 A Difference in Expectations and Framing

One difference between the policy processes for SMC and IPTi was in the researchers' expectations of the policy process. As mentioned previously, in the IPTi Consortium funding proposal approved by the BMGF in 2003, the researchers had high expectations that results would be consistent, and knowledge transfer would be quick. Policy engagement was planned to take place alongside the process of generating evidence on IPTi. A strategy was devised which set out a clear schedule that in 2006, that is to say at the time of the first TEG meeting, the Consortium would have generated a substantive body of evidence on IPTi to inform a WHO policy recommendation in that year. By framing the value of their research and their own success as a Consortium around a quick policy recommendation by WHO, the IPTi Consortium put themselves, and by extension, the WHO‐GMP evidence advisory process, under significant pressure. One interviewee recalled:

*Now where the IPTi consortium went wrong was that there was this day which was called the “green line” where we all go to it with all our evidence, and then the policy decision to implement IPTi would be made, but of course the reality is that the evidence would be considered and then a decision for IPTi policy would be made. But it wasn't really figured out like that. It was figured out that the “green line” meant green for go, and IPTi would be recommended, and IPTi would be implemented. And I think that that was really the biggest error, [the] supposition that the data would support a decision to go ahead. – KI44*

Although similar policy engagement also took place alongside the process of generating evidence on SMC, that process was perceived to be more organic, for example, via informal (by WHO standards) meetings between SMC researchers, WHO, and national malaria programs in 2008. The SMC researchers were not part of a formal “SMC Consortium” with an overt agenda to achieve policy goals. One reason for this is that they might have learned lessons from observing the experience of global malaria colleagues in the IPTi Consortium, who were in the midst of repeated TEG reviews and tensions with WHO‐GMP at around the same time. In any case, SMC researchers did not appear to have high expectations of quick knowledge transfer, nor the pressure of self‐imposed “green lines” to contend with, which might have contributed to a less fraught policy process with relatively tempered expectations, despite consistently highly efficacious trial results.

#### 3.4.2 Conflicting Agendas

The IPTi Consortium was made up of actors from different institutions with different primary objectives ranging from a focus on science to a concern with delivering programs. One thing they did have in common was high expectations that IPTi knowledge transfer would be quick and uncomplicated.^28^ Unfortunately, perceptions of the IPTi Consortium and views of it having an overt agenda, appeared to affect the functioning of the advisory bodies involved. This led to the perception of two sides pitted against the other. One interviewee summarized:

*It was bad. Aggressive from some of the researchers, aggressive from some members of the BMGF, an aggressive push back from WHO, I've never seen anything like it before. Everyone seemed to rally on the two sides. –KI49*

There was also a tension within the research community. Some IPTi Consortium members were strongly committed to contributing to public health by clear engagement in the policy process. Others felt, however, that scientists had to stay neutral and research‐focused. Although these tensions were less of an issue within the SMC policy process, many SMC researchers also expressed similar views about the role of researchers:

*You try to make sure that the key people know about [your study results] and that's by having a meeting or a symposium. Taking that any further, I've always been on the side that investigators shouldn't become lobbyists, and that somebody else should do that. You may need a lobbyist, but those are different people, it shouldn't be the investigators who did the trials…they may be asked to help, but you shouldn't have one of the key investigators initiating that process. – KI29*

The perceived overt advocacy by some IPTi Consortium members may have contributed to undermining their legitimacy within the IPTi policy development process. This was a consistent reflection across the various groups of interviewees—funder, researcher, and WHO staff. One interviewee shared their perception of the tension between WHO‐GMP and the IPTi Consortium from that time:

*I think clearly a problem [was] that WHO perceived the IPTi Consortium as being a mixture of investigators and advocates, and without a clear separation of those. So they saw this group as putting evidence forward and advocating strongly for implementation, for adoption of policy and implementation of IPTi. In fact, I think, in some ways the Consortium was perceived more as advocates than as sort of independent, unbiased investigators and so that colors the way things are looked at. If you think these people are flogging something and they've got lots of biases, then surely their data is biased and they're not revealing … For example, they may not have done the studies well enough to be sure that there aren't adverse reactions. That was a big issue. You could ask “Really? Did you really set things up so you picked up the signals?” – KI23*

In comparison, the researchers who were part of the SMC studies were perceived to have played a more neutral role, which was seen to help maintain their legitimacy. For example, one interviewee reflected:

*Many people, including myself, perceived and liked that the [SMC researchers] behaved the way that you expect scientists should behave…they really saw the various sides and carefully looked at the various angles [of the research question]. –KI35*

Reflections like these were common; the lack of pressure and, as a result, conflict during the SMC policy development process was considered by many interviewees to be its positive defining feature, in contrast with IPTi and its seeming legitimacy undermining missteps.

A big perceived misstep was the creation of the IPTi Policy Platform, which was part of both the Consortium and WHO‐GMP.^29^ Many interviewees felt that WHO‐GMP should never have been part of the IPTi Consortium or home to its Policy Platform, as it was a conflict of interest and detracted from the legitimacy of the process and the independent “balancing act” that is a WHO policy recommendation. One interviewee shared what they perceived to be a valuable lesson learned:

*There was one WHO staff member who was put on the IPTi proposal as part of the Consortium. Later on, this wound up raising questions about whether one should have someone as part of a consortium who is part of the institution that will be judge and jury of the evidence being generated. Does that blur those lines too much? I have to say that I have probably changed my view of that over time. I remember at the time being indignant that how could WHO have agreed to be part of the consortium, and then later reversing its position and claiming that it was not right for WHO to play that role. Now that I have spent time at WHO, and understand the importance of the independence of that evidence making process, I now understand those concerns. And I think that it probably is not a good idea to have someone as part of a consortium who is part of the agency that is convening the evidence review process; some separation is necessary. It doesn't need to be a firewall. There can be a dialogue, but you can't have that person be part of the group. They need to be having regular exchanges with the group and helping to steer the sort of evidence base that's required, but not be implicated as part of that group. I think that is an important balancing act. –KI39*

Having an overt policy agenda was not a mistake repeated by the researchers for the SMC set of studies. In addition, here WHO‐GMP involvement was viewed positively; they were seen as a “hands on” partner, meeting again for informal consultations between 2009 and 2010 when SMC researchers were collectively preparing their dossier for evidence review by the TEG. This was not perceived to be a conflict of interest by WHO‐GMP. It was seen to be in everyone's interest to make the process smooth while still maintaining institutional integrity via independence and transparency. Having a clear and transparent evidence review process for SMC appeared to be quite important to many interviewees. One interviewee recalled:

*For IPTI, it did not seem like a clear process; it seemed a bit cloak and dagger, or that events were taking place in a smoky dark room. There was no transparency as to how the process was supposed to be conducted. For the review of SMC, the fact that the Malaria Policy Advisory Committee had been convened in a transparent way, that everyone was aware who was on it, that there was clear terms of reference for the committee, that the Director General had signed off on the process, I think gave a lot of credibility in advance to the process, which is really important. If people coming into an evidence review have no idea what to expect, no idea what the steps are going to be, no idea who ultimately is making those decisions, then I think the process is on the rocks before it even gets going. – KI44*

During the SMC policy development process, WHO‐GMP was able to fulfill its own ideal notion of structural and legitimate power, without having to defend itself against other actors as it felt pressured to do during the IPTi process. By maintaining its power during the SMC process, WHO‐GMP maintained its legitimacy as a global health policy actor, which might have helped maintain the legitimacy of the policy development process itself.

## 4 Discussion

Explaining the differences in the policy development processes between IPTi and SMC requires understanding a set of interacting factors related to features of the evidence base as well as features of the process by which it was brought to bear on policymaking. IPTi was introduced as an innovation that was pursued by a group of committed public health practitioners and researchers, and internally framed along the lines of a quick and linear process. The IPTi Consortium's proposal to BMGF included a clear schedule and a Policy Platform to facilitate the policy development process. Consortium members believed that more evidence delivered in a timely way would persuade policymakers to recommend IPTi. However, over time, this internal expectation and pressure to meet the deadlines they had set for themselves in their proposal to BMGF led to a breakdown in consensus and trust between actors, followed by delays in its policy development. In comparison, the SMC policy process was never viewed as a battle between the actors involved. Here the policy process was viewed as open, inclusive, and transparent, which was WHO‐GMP's intention of what a good policy process should look like when it formed MPAC.^35^ By learning from its experience with IPTi, and optimizing the design and function of its principal scientific advisory committee to better serve its institutional needs, WHO‐GMP was perceived as having strengthened its malaria policy development process.

What appears to have edged the SMC evidence base over the one for IPTi was that, ultimately, it was more relevant to the question being asked by the TEG, with its perceived value as an intervention being boosted by the size and potential impact of its protective efficacy, and the high consistency of the results across RCT sites. Although the reasons for this difference (the highly focused and similar transmission settings for SMC studies) can be explained, a pooled protective efficacy of 75% for SMC compared to 30% for IPTi made the potential impact of SMC a difficult policy option to ignore. In other words, while the results of the RCTs for IPTi would be considered “credible” by standard evidence hierarchy measures, and comparable to other preventive malaria interventions, the evidence base for SMC compared to IPTi was perceived to be both “credible” and “salient”, which contributed to making it appear better, or more appropriate for policy consideration.

The study findings also suggest that the breakdown in consensus and trust in the policy process, due to the different expectations, conflicting agendas, and in some instances, the overt advocacy of the actors involved, might have contributed to the perception of problems that undermined the policy development process for IPTi, in comparison to SMC. The contestation around the IPTi policy process might have contributed to negative perceptions of its policy value. Contestation, as a form of deliberation and consensus building, is not necessarily a “bad” thing, particularly when built into institutional arrangements that aim to improve the legitimacy of governing processes through deliberation and inclusion of multiple views.^45^ Some scholars have seen the need for deliberation as particularly important when public policy relies on delegation to scientific experts that serve to provide scientific advice.^46^ Institutional approaches in the policy sciences recognize that institutions can be thought of in terms of formal structures, and also as rules that shape how decisions are made.^47^ In the case of SMC, although there was not necessarily as much deliberation over the evidence as there was for IPTi, it appears that having clear expectations from all sides of the evidence advisory process, with a clear structure and terms of reference for MPAC members, as well as transparency of the evidence consideration, might have led to the process for SMC appearing more “legitimate” to those involved in it evidence advice and policy development.

These findings are not meant to imply that one evidence base was stronger or weaker than the other was, or that the process of evidence use is necessarily more important than features of the evidence itself. Indeed, both feature in important but differing ways. As such, these findings help to reinforce how the factors of credibility, salience, and legitimacy all appear to influence evidence use, with particular insights into an agency with a particular technical remit and expert body of stakeholders informing global health policy making.

While these findings emerge from a pair of specific malaria policy developments, there may be reasons to believe similar issues would be relevant elsewhere. Indeed, the issues of credibility, salience, and legitimacy derived from a very different study conducted on sustainable development related to concerns of the lay public as well as of scientists. Thus seeing similar issues arise in a technical body made up of individuals with broadly similar scientific training helps to illustrate that even in these groups, features outside scientific quality can matter when it comes to evidence use for policy and planning.

What might be an important additional factor for WHO however, as it continues to improve its internal guideline development processes,^4^ is to consider more specifically the processes followed by its advisory bodies, in addition to concern over how to judge or rank evidence. Specifically considering how to build evidence advisory structures that are open, inclusive, and transparent might serve to promote the legitimacy of its policy decisions and decrease potential conflicts of interests in a global health‐funding environment where private funders are viewed as having increasing influence on the global health agenda.^48^ For example, the BMGF are already one of the largest donors to the WHO, and within the field of global malaria, they funded both the IPTi Consortium and many of the SMC studies, in addition to the policy‐strengthening grant that led to the creation of MPAC.[[qv: 28,35,37,48f,49]] There is very little in the world of global malaria control and elimination that is not funded or at least influenced by BMGF.^50^ While many in the global malaria community are quick to point out the positive outcomes of what funding coming from the BMGF can achieve,[[qv: 48e]] the findings here illustrate the importance of process legitimacy in addition to concern over outputs and outcomes.

## 5 Conclusion

In the case of the policy development processes for IPTi and SMC, the findings show that “good evidence” from a purely technical (credibility) perspective was not sufficient to ensure universal agreement and uptake of recommendations, even within a highly technocratic body such as the WHO‐GMP. The findings suggest that evidence also needed to be relevant (salient) to the policy question being asked, and technical actors retained a concern over the legitimacy of the process by which technical evidence was brought to bear in the policy development process. Cash and colleagues findings from the field of sustainable development,^18^ that evidence must be credible, salient, and legitimate to be accepted by the public, appears to equally apply within scientific advisory committees, albeit nuanced by their specific contextual realities.

While the WHO has principally focused on technical criteria for evidence inclusion in its policy and guideline development processes, the study of the MPAC suggests that the design and functionality of its scientific advisory committees might also have a role to play within its overarching evidence advisory system. Scientific advisory committees should consider enabling transparent, responsive, and credible processes of evidence review, to ensure that they are effective in producing advice that ultimately leads to policy recommendations by WHO. Such legitimacy may also be important to implementation of recommendations by WHO member states, particularly considering the current funding environment in which WHO is highly reliant on external sources of funding, both for programmatic work, as well as for funding research that aims to ultimately inform policy and practice.

## Conflict of Interest

The authors declare no conflict of interest.
